# Integrative Pathway-Centric Modeling of Ventricular Dysfunction after Myocardial Infarction

**DOI:** 10.1371/journal.pone.0009661

**Published:** 2010-03-11

**Authors:** Francisco Azuaje, Yvan Devaux, Daniel R. Wagner

**Affiliations:** 1 Laboratory of Cardiovascular Research, Centre de Recherche Public - Santé, Luxembourg, Luxembourg; 2 Division of Cardiology, Centre Hospitalier, Luxembourg, Luxembourg; Fondazione Telethon, Italy

## Abstract

**Background:**

A significant proportion of myocardial infarction (MI) patients undergo complex, coordinated perturbations at the molecular level that may eventually drive the occurrence of ventricular dysfunction and heart failure. Despite advances in the elucidation of key processes implicated in this condition, traditional methods relying on gene expression data and the identification of individual biomarkers in isolation pose major limitations not only for improving prediction power, but also for model interpretability. Mechanisms underlying clinical responses after MI remain elusive and there is no biomarker with the capacity to accurately predict ventricular dysfunction after MI. This calls for the exploration of system-level modeling of ventricular dysfunction in post-MI patients. Within this discovery framework key perturbations and predictive patterns are characterized by the integrated biological activity levels observed in pathways, rather than in individual genes.

**Methodology/Principal Findings:**

Here we report an integrative approach to identifying pathways related with ventricular dysfunction post MI with potential prognostic and therapeutic value. We found that a diversity of pathway-level perturbations can be profiled in samples of patients with ventricular dysfunction post MI, most of which represent major reductions of gene expression. Highly perturbed pathways included those implicated in antigen-dependent B-cell activation and the synthesis of leucine. By analyzing patient-specific samples encoded with information derived from highly-perturbed pathways, it is possible to visualize differential prognostic patterns and to perform computational classification of patients with areas under the receiver operating characteristic curve above 0.75. We also demonstrate how the integration of the outcomes generated by different pathway-based analysis models may improve ventricular dysfunction prediction performance.

**Significance:**

This research offers an alternative, comprehensive view of key relationships and perturbations that may trigger the emergence or prevention of ventricular dysfunction post-MI.

## Introduction

Heart failure (HF) is a clinical condition that can be defined as the heart's inability to pump enough blood to meet physiological requirements. HF is caused by cardiac disease via the interplay of multiple molecular and environmental factors. The major cause of HF is ventricular dysfunction after myocardial infarction (MI). Post-MI patients undergo molecular alterations that result in structural and functional adaptations in the heart, and which in turn may eventually trigger the occurrence of ventricular dysfunction and HF [1]. Despite advances in molecular and medical research, mortality and morbidity of HF after MI remain unacceptably high [2], [3]. It has to be acknowledged that key mechanisms underlying clinical responses after MI remain elusive. Furthermore, currently there is no biomarker with the capacity to accurately predict ventricular dysfunction after MI, with the exception of brain natriuretic peptide [4]. Thus, a crucial goal is to discover knowledge to predict the onset of ventricular dysfunction after MI.

A major challenge for translational biomedical research in the “post-genome era” is to disentangle the complexity of multiple levels of “omic” information, which can be used to improve our understanding of the functioning (or malfunctioning) of biological events implicated in the development of disease. Another important requirement is to develop computational methodologies for facilitating the interpretation of large-scale experiments and the prediction of clinical outcomes. Although it is known that (post-MI) ventricular dysfunction is the by-product of the large-scale, dynamic interaction of complex molecular systems, there is a lack of understanding of systemic mechanisms that can aid in the prediction and treatment of this disease.

In the past two decades, large-scale gene expression analysis has significantly contributed to the identification of possible processes implicated in different disease domains. However, the use of genome-wide microarrays to identify new biomarkers is still in its infancy especially in the cardiovascular domain. Very few studies reported the use of transcriptomic profiling to aid in the search of new cardiac biomarkers [5], most likely because of the unavailability of cardiac tissue. In an interesting study published by Wingrove et al. [6], gene expression of peripheral blood cells was found to be correlated with the severity of coronary artery disease, opening up new avenues for the search of cardiovascular biomarkers. Indeed, the possibility to use transcriptomic biosignatures of blood cells to predict clinical outcome after MI is particularly attractive.

The standard approach to biomarker discovery based on gene expression data typically involves two main steps: a) detection of differentially expressed genes, and b) the description of the resulting sets of genes in terms of their involvement in specific biological processes. The former is commonly achieved with different (univariate) statistical testing procedures. The latter can be done by estimating the statistical “enrichment” of standard functional annotations in the set of genes, such as those defined in the Gene Ontology (GO) and the KEGG pathways databases [7]. Despite its proven utility for guiding biomarker research, this standard approach presents different limitations related to both the accuracy and interpretability of the resulting predictive models [8].

Previous research suggests, for example, that biomarkers discovered under this traditional framework may: a) be difficult to reproduce using independent datasets, b) lack predictive robustness when evaluated with different computational prediction model and datasets, c) be more difficult to interpret in the context of previous and emerging evidence [9], [10], [11].

This may be explained in part by the possibility that highly-differentially expressed genes encode “downstream effectors” or “reflectors” of biological malfunction, which tend to incorporate elevated levels of both biological and experimental noise. Furthermore, it has been suggested that an emphasis on the detection of highly-differentially expressed genes may constrain the identification of genes with potential causal roles in disease [12]. This encourages the development of alternative, systems-based methodologies that can both enhance and complement the predictive power and interpretability of traditional approaches.

To address these concerns, bioinformatic advances for biomarker discovery have involved the detection of differentially expressed genes in the context of biological pathways or using network-derived predictive features [9], [10], [11]. These methods allow researchers to identify genes that may not be necessarily highly-differentially expressed across control-case groups, but whose integration into computational predictive models can improve prognostic accuracy. Moreover, these methods offer a more descriptive, and possibly less biased, approach to visualizing and explaining these predictions. For example, the Gene Set Enrichment Analysis (GSEA) proposed by Subramanian et al. [9] and subsequent versions of this technique [13], enable researchers to identify lists of genes that can be used to distinguish between control and case samples based on the expression levels that these genes show in previously-annotated sets of interrelated genes, i.e. canonical pathways. Several modeling approaches to the progression and recurrence of cancer have been recently proposed based on pathway-based signatures [10], [11], [12]. For instance, instead of detecting potential biomarkers as individual genes, Chuang et al. [10] implemented a methodology in which new breast cancer biomarkers were represented as sub-networks obtained from protein interaction databases. Lee et al. [11] identified condition-responsive genes, whose combined expression measurements can be used to perform accurate phenotype classification. These biomarker discovery methodologies are based on the idea of finding statistically-detectable differences between phenotypes using patterns of gene expression observed in specific molecular pathways.

Although one may further argue in favor of these approaches, there is no unique or widely-accepted solution for implementing pathway-driven biomarker discovery. In addition, these techniques can represent alternative solutions that in combination may enhance our understanding of mechanisms underlying the behavior of potential biomarkers and therapeutic targets. In the area of cardiovascular diseases, in general, and in HF research, in particular, this type of investigations have not been sufficiently investigated [8], [14], [15].

Here we report the integrated analysis of gene expression data and molecular pathways relevant to ventricular dysfunction after MI. We aimed to characterize this condition with regard to perturbations observed in hundreds of pathways, and to explore the application of this knowledge for automated prognostic tasks. To accomplish these goals, we implemented an alternative pathway-centric approach to identifying biomarkers and performing patient-specific classification, which differs from the techniques introduced above in a number of ways. Such differences refer to the way pathways are quantitatively described, ranked and applied to perform patient-derived sample classification (Methods and Discussion). Furthermore, we were interested in exploring the potential descriptive and predictive complementarities that can be brought with the proposed approach and a previously-published method. To address this issue, we compared and integrated the predictions made by our method with those generated by GSEA.

## Methods

### Research framework

**Figure 1:**
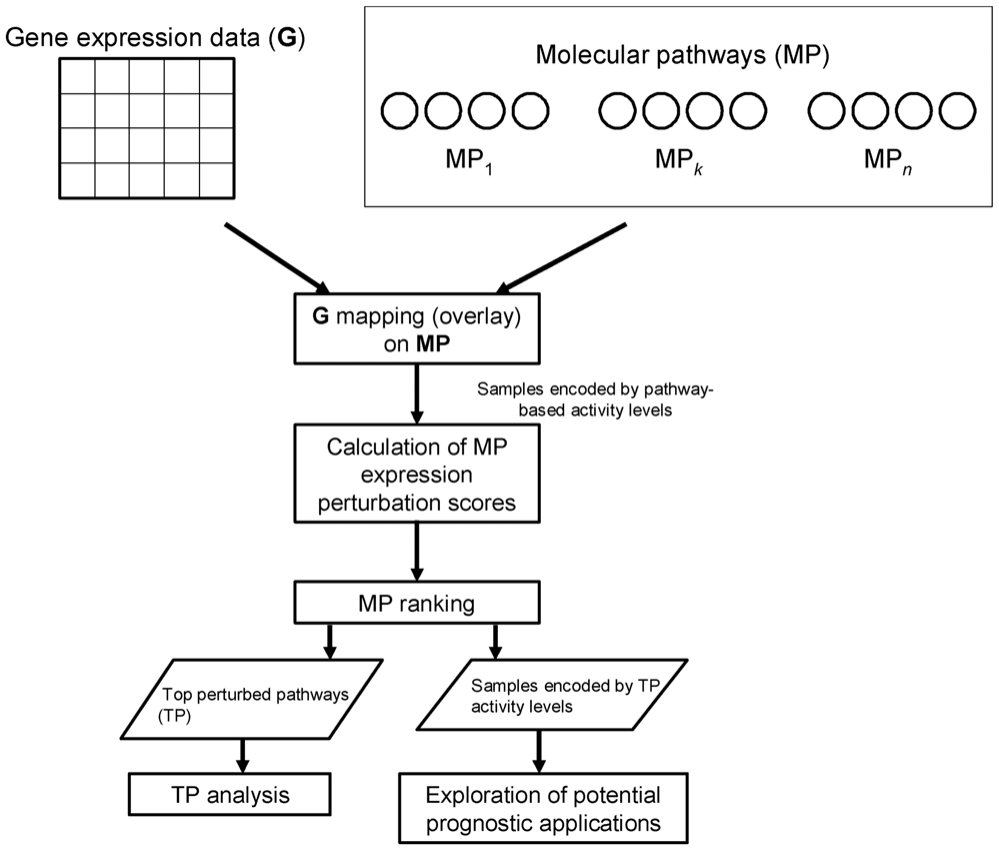
Overview of research pipeline and main outcomes. Integrative pathway-based modeling of ventricular dysfunction after MI.

In this study we searched for validated biological pathways that show major gene expression perturbations in ventricular dysfunction. We compared the “level of activity” of pathways on the basis of gene expression measurements derived from whole-blood samples in patients with ventricular dysfunction (VD+, ejection fraction ≤40%) and in patients without ventricular dysfunction (VD−, ejection fraction >40%) after MI. This represents an integrated analysis framework, which comprises the combined processing of both types of information in parallel. Figure 1 summarizes the research pipeline implemented and main outcomes obtained.

The first analytical phase involved the selection and transformation of gene expression and molecular pathways. The gene expression dataset, **G**, consisted of 32 samples originating from 16 patients with ventricular dysfunction and from 16 patients without ventricular dysfunction post MI. A set of 639 curated molecular pathways, **MP**, were analyzed. Additional information on these datasets is provided below. Each sample, ***s***_i_, was encoded as a vector of more than 15,000 gene expression values together with their corresponding class labels. The expression measurements in each sample, ***s***_i_, were mapped (overlaid) onto each pathway in **MP**. This resulted in 20448 (32×639) pathways reflecting the specific gene expression levels in the different samples, i.e. each sample was represented by the expression levels observed in 639 pathways. Of these pathways, only 637 included at least one gene with an expression value in **G**. Thus, after the sample-pathway mapping, 20384 (32×637) sample-specific pathways were available for further analyses.

The activity level, hereafter referred to as *L* values, of each sample-specific pathway and a global “perturbation score”, *psc*, for each pathway were estimated. A *psc* is computed by comparing the *L* values across the VD+ and VD− samples, as explained in the next sub-section. The *psc* values provide a quantitative indication of the alteration of gene expression in the pathways of VD+ samples in relation to VD− samples, or vice versa. Once the *psc* values were estimated, we ranked the 637 pathways to identify the pathways exhibiting the largest gene expression perturbations in VD+, from now on also referred to as “top pathways” (TP). Subsequent analyses focused on these TP, which were functionally characterized and can be seen as potential key mechanisms underlying the phenotypic differences investigated here. Moreover, our hypothesis is that these pathways may also provide the basis for prognostic applications. We compared these predictions with those made by an alternative, published methodology (more details below).

We explored potential prognostic applications based on the analysis of *L* values that represent the samples obtained from the VD+ and VD− patients. Different classification models were implemented and compared with those obtained from a well-known gene expression analysis technique (see below). In a final analysis phase, we assessed the integration of these methodologies to support the discovery of potential novel biomarkers and more powerful clinical decision-making support.

### Detecting and scoring pathway perturbations in ventricular dysfunction

**Figure 2:**
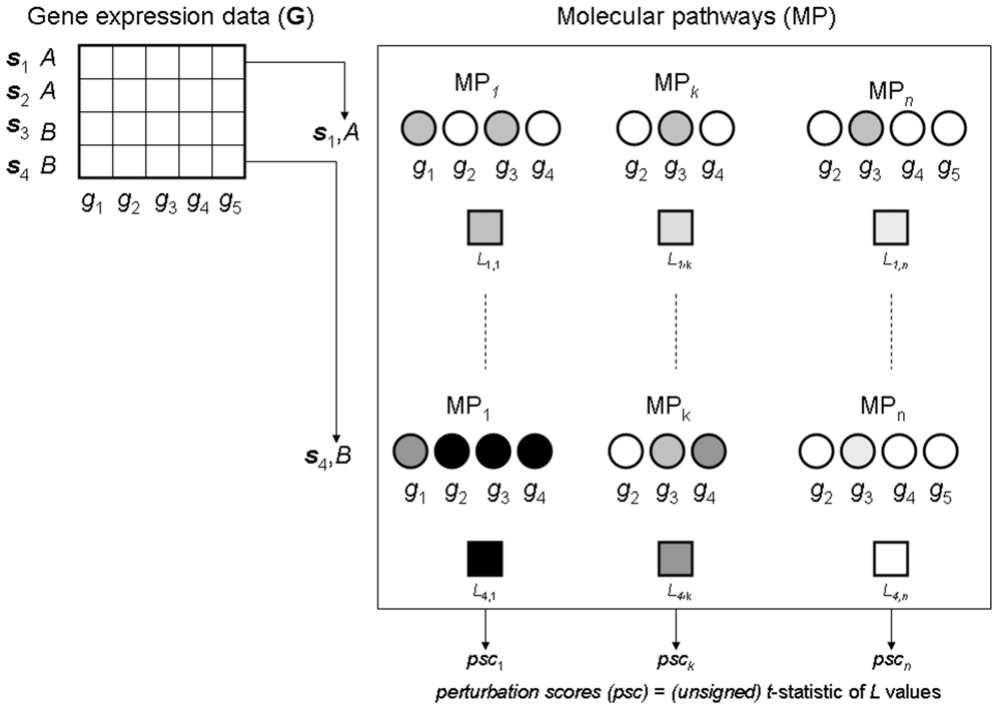
Main algorithmic steps and outcomes in pathway perturbation detection. Identification of molecular pathways differentially perturbed in VD+ and VD− patients. The calculation of the *psc* values of 3 hypothetical pathways, MP, is illustrated here with 4 samples, **s**. These samples belong to two hypothetical clinical classes or phenotypes, *A* and *B*. The *psc* value assigned to a pathway is computed with the *t*-statistic obtained from the pathway's activity levels, *L*, observed in the different samples. For a given sample, an *L* value is defined as the mean expression value observed in a pathway. For example, *L*_4,*k*_ represents the activity level of the *k* ^th^ pathway, MP*_k_,* in relation to sample **s**_4_. Sample-specific expression and *L* values are here shown for **s**_1_ and **s**_4_ only, dotted lines represent the samples not shown here for clarity. For a given pathway, shaded circles are used to represent the intensity of the expression measurements in each sample. Shaded squares reflect the magnitude of *L* values obtained in a given pathway in relation to a specific sample.

Figure 2 depicts the main algorithmic steps and outcomes required to calculate *L* and *psc* values. This is illustrated with an example including 3 hypothetical pathways and 4 samples, which belong to two clinical classes, *A* and *B*. For a given sample, ***s***_i_, a *L_i,k_* value is calculated with respect to each pathway, MP*_k_*. The pathways consist of sets of genes, *g*, which are graphically represented with circles in Figure 2. Shaded circles are used to indicate the intensity of the expression measurements in each sample. *L_i,k_* is obtained by calculating the mean expression value observed in MP*_k_* for the given sample, ***s***_i_. These calculations are performed for all samples and pathways. Figure 2 portraits *L* values with colored squares to reflect the magnitude of these values. At the end of this procedure, each sample will be associated with *n L*-values, and each pathway will assigned to *m L*-values, with *n* and *m* representing the total number of pathways and number of samples respectively. In our experiments *n* = 637 and *m* = 32. The next step was to calculate *psc* values for each pathway using their corresponding *L*-values. The *psc* value of a pathway, MP*_k_*, is equal to the unsigned *t*-statistic value obtained when comparing the *L* values observed across the two investigated classes, *A* and *B*. Thus, *psc* estimates the magnitude of the differential change in expression observed in MP*_k_* for the given gene expression dataset, **G**.

For each pathway *psc* value, *P* values were calculated to estimate the statistical significance of these scores. This was done by implementing a permutation-based testing procedure. The observed *psc* values were independently compared with a null-distribution of *psc*_null_ values. A single *psc*_null_ refers to the perturbation scores measured in a “permuted dataset”. A permuted dataset is constructed by randomly permuting the class labels in the original gene expression dataset. The null-distribution was approximated by creating 10,000 permuted datasets and computing their corresponding *psc*_null_ values. A *P* value reflects the proportion of *psc*_null_ values expected to be as large (or larger) as the observed *psc* value. Thus, the lower a *P* value, the higher the confidence that can be assigned to the observed *psc* value.

### Computational prognosis applications

We explored the potential application of pathway-derived information for supporting ventricular dysfunction prognosis. We first represented patient-specific samples using their pathway activity levels, *L*, corresponding to the top 10 perturbed pathways, TP. This information allowed us to train and test machine learning classification models. The results reported here represent the best classification performances, which were obtained with an instance-based learning classification model, *KStar* [16], and estimated with leave-one-out cross-validation (LOOCV).

To further assess the relevance of the proposed approach, we studied classification models based on genes that were detected by GSEA as significantly differentially expressed [9]. Finally, we assessed the predictive integration of different potential biomarkers identified by our approach and GSEA.

### Ethics Statement

The protocol was approved by the local ethics committee (Comité national d'éthique de la recherché, CNER) and written informed consent was obtained from all patients.

### Patients and gene expression data

Patients with acute MI enrolled in a national MI registry and treated with primary percutaneous coronary intervention were used in this study. Acute MI was defined by the presence of chest pain <12 hours with significant ST elevation and increase in creatine kinase and troponin I to greater than 2-fold upper limit of normal. Blood samples were obtained at the time of mechanical reperfusion in PAXgene™ tubes. Ejection fraction (EF) measured with echocardiography using Simpson's method 1 month after MI was used to classify the patients into low EF (≤40%; 33±7%) or high EF (>40%; 61±10%) groups. Both groups of patients were age- and sex-matched and did not differ with respect to reperfusion time, final coronary flow, multivessel disease, history of previous infarction, cardiovascular risk factors or treatment. As expected, enzyme release was higher in the group of patients with impaired EF consistent with impaired tissue perfusion. Of note, 6 patients in the VD+ group died (37%) and 3 developed heart failure during 2 year follow-up while no patient died in the VD− group and only 1 developed heart failure. These data confirm the poor prognosis associated with ventricular dysfunction after MI.

Total RNA from the 32 MI patients was extracted from whole blood cells by the PAXgene™ technology. Transcriptomic profiles were obtained using oligonucleotide microarrays representing 25,000 genes [17]. All data are MIAME-compliant and available at the Gene Expression Omnibus database (www.ncbi.nlm.nih.gov/geo/) under the accession number GSE11947.

### Information resources and software tools

The collection of 639 pathways were obtained from the Molecular Signatures Database (MSigDB) hosted at the Broad Institute [9]. We focused on the “C2 collection” of canonical pathways, which represent curated metabolic and signalling pathways originating from different online databases. The algorithms proposed above were implemented in our laboratory with the Java programming language. Statistical displays were created with the Statistica package [18]. Pathways were described in terms of statistically detectable Gene Ontology (GO) biological process and cellular localization terms, and were obtained with the Fatigo tool, under the BABELOMICS (v3.1) software platform [7]. The *P* values describing the statistical significance of the overrepresentation of GO terms in the pathways were estimated with (two-tailed) Fisher's exact tests and corrected to account for multiple-hypotheses testing using the Benjamini & Hochberg adjustment procedure [7]. Unless otherwise indicated, only corrected *P* values are reported here and statistical significance is defined at the level of *P* = 0.05. Clustering analysis and visualisation were implemented with the GEPAS (v4.0) platform [19]. Supervised classification models were implemented with the Weka software system [20].

## Results

### Landscape of perturbed molecular pathways in post-MI ventricular dysfunction

**Figure 3:**
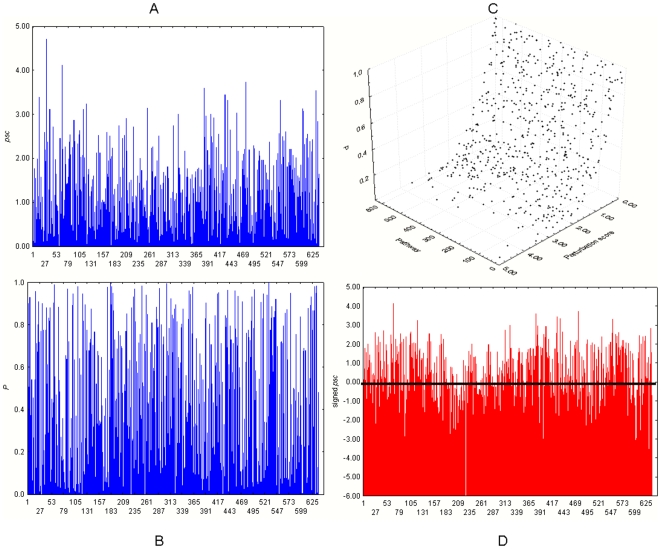
Landscape of perturbed molecular pathways in post-MI ventricular dysfunction. In A, B, D vertical bars represent the magnitude of the perturbation score for a given pathway. A: Overview of perturbation scores, *psc*, (vertical axis) across 637 molecular pathways (horizontal axis). B: Overview of *P* values (vertical axis) for the perturbation scores shown in A. C: 3D display with the distribution of molecular pathways, perturbation scores and *P* values. D: Display of perturbation scores across 637 molecular pathways together with the direction of the perturbation in relation to patients with ventricular dysfunction: The region below the horizontal (black) line includes pathways that exhibit a reduction of their (mean) gene expression.

The *psc* of 637 molecular pathways were calculated using the gene expression data from VD+ and VD− samples. Figure 3 provides global views of the results. In panels A, B and D, vertical bars represent the magnitude of the *psc* for a given pathway. The set of *psc* ranges from values near 0 to 4.70 (Figure 3A). Figure 3B shows the corresponding *P* values associated with the *psc*. Figure 3C presents a 3D display of the complete set of *psc* and *P* values, and indicates that the strength of the observed perturbations is directly proportional to their statistical significance levels. Figure 3D, a version of Figure 3A, displays the *psc* across the 637 molecular pathways together with the direction of the perturbation in relation to VD+ patients, i.e. it reflects whether the perturbation represents either an increase or decrease in gene expression in the VD+ class. This was done by including the signed version of the *psc*, i.e. the observed *t*-statistic assigned to each pathway. In this panel, the region below the horizontal (black) line includes pathways that exhibit a reduction of their (mean) gene expression.

### Major pathway perturbations and dependencies in post-MI ventricular dysfunction

To assess the potential biological relevance of the pathway-specific perturbations, we concentrated on the analysis of the pathway showing the largest perturbations in the VD+ samples. Table 1 summarizes the top 10 pathways exhibiting the strongest perturbations. The pathways are named with the standard name given in the MSigDB. From now on, we will refer to these pathways as top-perturbed (TP) pathways. The pathway with the strongest perturbation is the ASBCELLPATHWAY, a pathway responsible for antigen dependent B- cell activation. Among these pathways found to be significantly de-regulated are: CCR3PATHWAY (CCR3 signaling in eosinophils), HYPERTROPHY_MODEL(a pathway implicated in time- and exercise-dependent gene regulation in human skeletal muscle) [21]; and the Erk1/Erk2 Mapk Signaling pathway (ERKPATHWAY). Table S1 offers a more detailed functional characterization of these pathways, including the type of gene expression change observed in relation to VD+ samples.

**Table 1:** Top 10 molecular pathways exhibiting the largest gene expression perturbations in VD+ samples.

TP	Name	*psc*	*P*	Genes
TP1	ASBCELLPATHWAY	4.70	1.0E-4	CD28 CD4 CD80 HLA-DRA HLA-DRB1 IL10 IL2 IL4 TNFRSF5 TNFRSF6 TNFSF5 TNFSF6
TP2	CCR3PATHWAY	4.12	2.0E-4	ARHA CCL11 CCR3 CFL1 GNAQ GNAS GNB1 GNGT1 HRAS LIMK1 MAP2K1 MAPK3 MYL2 NOX1 PIK3C2G PLCB1 PPP1R12B PRKCA PRKCB1 PTK2 RAF1 ROCK2 MAPK1
TP3	PELP1PATHWAY	3.73	9.0E-4	CREBBP EP300 ESR1 MAPK1 MAPK3 PELP1 SRC
TP4	HYPERTROPHY_MODEL	3.60	9.0E-4	ANKRD1 ATF3 CYR61 DUSP14 EIF4E EIF4EBP1 GDF8 HBEGF IFNG IFRD1 IL18 IL1A IL1R1 JUND MYOG NR4A3 TCF8 VEGF WDR1
TP5	VALINE_LEUCINE_AND_ISOLEUCINE_BIOSYNTHESIS	3.55	0.0010	IARS LARS LARS2 PDHA1 PDHA2 PDHB
TP6	METPATHWAY	3.44	0.0020	ACTA1 CRK CRKL DOCK1 ELK1 FOS GAB1 GRB2 GRF2 HGF HRAS ITGA1 ITGB1 JUN MAP2K1 MAP2K2 MAP4K1 MAPK1 MAPK3 MAPK8 MET PAK1 PIK3CA PIK3R1 PTEN PTK2 PTK2B PTPN11 PXN RAF1 RAP1A RAP1B RASA1 SOS1 SRC STAT3
TP7	ALANINE_AND_ASPARTATE_METABOLISM	3.39	0.0026	ABAT ADSL ADSS AGXT AGXT2 ASL ASNS ASPA ASS CAD CRAT DARS DDO GAD1 GAD2 GOT1 GOT2 GPT GPT2 NARS PC
TP8	MPRPATHWAY	3.32	0.0025	ACTA1 ADCY1 CAP1 CCNB1 CDC2 CDC25C GNAI1 GNAS GNB1 GNGT1 HRAS MAPK1 MAPK3 MYT1 PIN1 PRKACB PRKACG PRKAR1A PRKAR1B PRKAR2A PRKAR2B RPS6KA1 SRC
TP9	SIG_REGULATION_OF_THE_ACTIN_CYTOSKELETON _BY_RHO_GTPASES	3.32	0.0025	ACTG1 ACTG2 ACTR2 ACTR3 AKT1 ANGPTL2 CDC42 CFL1 CFL2 FLNA FLNC FSCN1 FSCN2 FSCN3 GDI1 GDI2 LIMK1 MYH2 MYLK MYLK2 PAK1 PAK2 PAK3 PAK4 PAK6 PAK7 PFN1 PFN2 RHO ROCK1 ROCK2 RPS4X VASP WASF1 WASL
TP10	ERKPATHWAY	3.23	0.0027	DPM2 EGFR ELK1 GNAS GNB1 GNGT1 GRB2 HRAS IGF1R ITGB1 KLK2 MAP2K1 MAP2K2 MAPK1 MAPK3 MKNK1 MKNK2 MYC NGFB NGFR PDGFRA PPP2CA PTPRR RAF1 RPS6KA1 RPS6KA5 SHC1 SOS1 SRC STAT3

*psc*: perturbation scores. *P*: Statistical significance of the perturbation. TP: Top pathway number.

**Figure 4:**
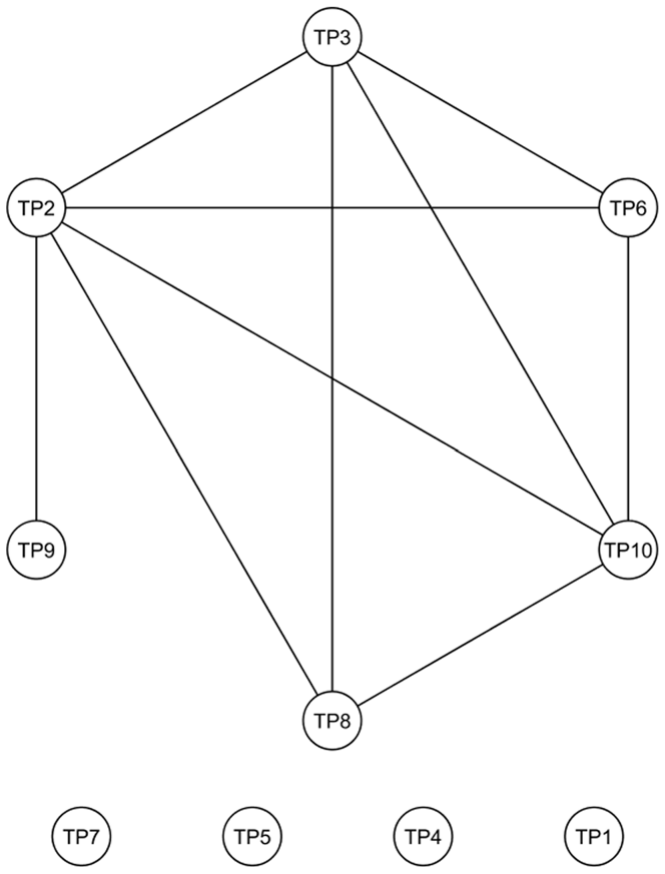
Interactions across perturbed pathways. Dependencies between the top 10 molecular pathways exhibiting the largest gene expression perturbations in VD+ samples. Nodes depict the top 10 molecular pathways exhibiting the largest gene expression perturbations in VD+ samples, as described in Table 1. Arcs linking the nodes are used to indicate functional relationships between these pathways, i.e., proteins are shared between any two pathways. TP1, TP4, TP5 and TP7 do not share proteins with other pathways.

These pathways exhibit various functional dependencies with regard to shared biological processes and cellular localizations. Moreover, they functionally interrelate through proteins shared by the different pathways. These relationships are depicted in Figure 4. Arcs are used to indicate that any two pathways share proteins. This illustrates the coordinated and complex functional inter-play required to drive specific processes relevant in post-MI ventricular dysfunction.

### Pathway-specific activity levels distinguish patient clinical outcomes

The results reported above represent system-level insights into some of the mechanisms possibly underpinning the development of ventricular dysfunction in post-MI patients. Motivated by the identification of pathways displaying substantial gene expression perturbations, we explored the application of TP activity levels to represent patient-specific samples and to distinguish between them. As a starting point, each sample was encoded with the activity levels observed in each of the top 10 pathways described in Table 1. Under this scheme, rather than using gene expression values, each patient can be represented by 10 pathway-specific activity levels, *L* values, whose calculation was described in Methods (Figure 2).

**Figure 5:**
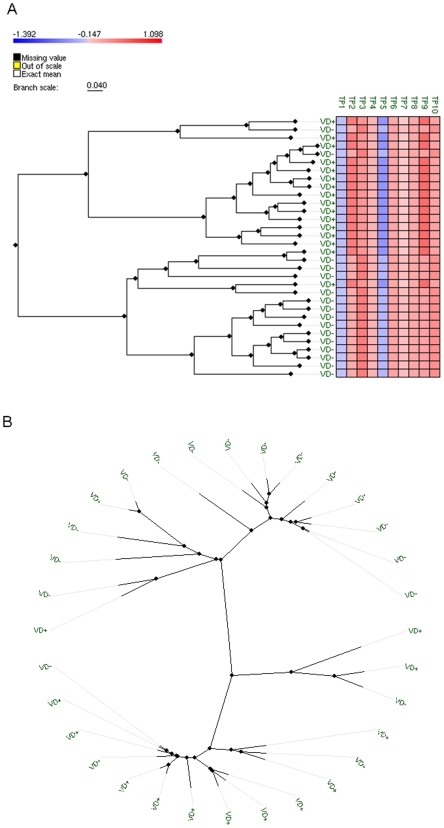
Grouping of samples based on perturbed pathways. Hierarchical clustering of VD+ and VD- samples based on the activity levels of the top 10 perturbed pathways. Each sample was encoded using its corresponding (ten) pathway activity levels. A pathway activity level for a given sample is defined as the mean expression value observed in the pathway for the sample under consideration. A: clustering visualization using a dendrogram. B: clustering visualization using an unrooted tree. The length of the branches reflects pair-wise distances between samples.

Using this sample-encoding scheme, we implemented a hierarchical clustering of the samples. Note that this procedure does not incorporate information about the class (VD+ and VD−) assigned to each sample, i.e. the clustering represents an unsupervised classification of samples. Figure 5 presents alternative displays of the resulting clustering based on the activity levels of the top 10 perturbed pathways. The true class labels for each sample are displayed. In this figure, panels A and B offer clustering visualizations using a dendrogram and an unrooted tree respectively. The length of the branches in these graphs reflects the pair-wise distances between samples. These results showed that the samples can be discriminated in terms of clinical outcome. Same-class patients are clustered together, and dissimilar samples are separated. The clustering visualization reveals the existence of two major groups of patients: One grouping 14 (out of 16) VD+ samples, and the other including 14 (out of 16) VD− samples. These results suggest that pathway-specific activity levels may be a useful visualization-driven, exploratory approach to clinical decision-making support.

### A pathway-based prognosis application

The unsupervised discriminatory capability of *L* values shown above encourages the idea of assessing the potential application of this information for (supervised) prognosis tasks. The *L* values in each sample were used as inputs to different classification models, which were evaluated with LOOCV. The best performances for a single-*L* input were observed when encoding the samples with the *L* values observed in TP1 and TP5, which are the pathways involved in antigen dependent B-cell activation and biosynthesis of valine, leucine and isoleucine (Table 1). Using the *L* values from these pathways and models based on the KStar classifier, a maximum AUC (area under the receiver operating characteristic curve) value of 0.80 was obtained. Combinations of multiple *L* values into integrated classification models, in general, degraded classification performance. This indicates that these pathways contribute redundant (correlated) information for sample classification. However, this did not hold true for one integrated biomarker model. The combination of the *L* values from TP1 and TP5 improved single-input classification performance (AUC = 0.84).

### TP1-specific gene expression profiles

The significant perturbation observed in TP1 motivated us to examine the expression profiles of individual genes (and combinations) in this pathway and to explore their application in prediction models. Independent classification models were built using these individual expression profiles as inputs. Classification models using all measured TP1 genes as individual inputs reported AUC values below 0.70. The best classification performance was obtained with models based on genes CD28 and CD80 together (AUC = 0.72, KStar classifier). These results underscore the prediction power of integrated pathway markers (*L* values) in comparison to standard models based on individual gene profiles, including those derived from highly perturbed pathways.

### Alternative pathway perturbation analysis and biomarker identification

To further illustrate the potential prognostic application of pathway *L* values, we implemented classification models built on genes identified by a published methodology for gene expression analysis, and compared the resulting prediction performances against the results reported above. This task was carried out with the GSEA algorithm [9]. This technique not only allows the identification of differentially expressed genes between the sample categories, but also it reports in which pathways such perturbations occur.

Using the same set of (637) pathways analysed above and the 32 (VD+ vs. VD−) samples, GSEA found significant gene expression perturbations in pathways that were included by our approach in the list of top 10 perturbed pathways (Table S2). For example, GSEA and our approach identified ERKPATHWAY, CCR3PATHWAY, METPATHWAY and MPRPATHWAY in the top 10 list of molecular perturbations. In addition, other pathways were highlighted by GSEA which were not among our top perturbed pathways. For example, GSEA found that the strongest perturbation is observed in a set of 47 genes defining a CIRCADIAN_EXERCISE pathway as defined in the MSigDB (Subramanian et al., 2005). These results illustrate not only the consistency of these two approaches, but also, and maybe more importantly, their complementarity.

The 10 most highly differentially expressed genes detected by GSEA were: C20orf20, QSOX1, HK2, EIF2AK2, LMNB1, TP53RK, LY9, CCDC107, PAQR8, and SLC37A4. The following genes showed increased expression in VD+ samples relative to VD− samples: C20orf20, QSOX1, HK2, EIF2AK2, and LMNB1. The other highly differentially expressed genes showed reduced expression levels in VD+ samples. Different single-gene and multiple-gene classification models were built. With regard to single-gene models, the best classification performances were obtained with C20orf20 (AUC = 0.85) and TP53RK (AUC = 0.85) using a KStar classifier and LOOCV. Integrated classification models combining sub-sets of genes, in general, degraded classification performance. The combination of TP53RK and LMB1 reported a maximum AUC of 0.82.

### Integrated biomarker models increase prognostic performance

The previous sections indicate that these techniques provide alternative, yet potentially complementary, approaches to biomarker discovery. This motivated us to investigate possible integrated classification strategies, which combined input features derived from our pathway-based VD+ model (*L* values) and from the GSEA method (highly differentially expressed genes). We found that different biomarker combination schemes can enhance classification performance.

**Figure 6:**
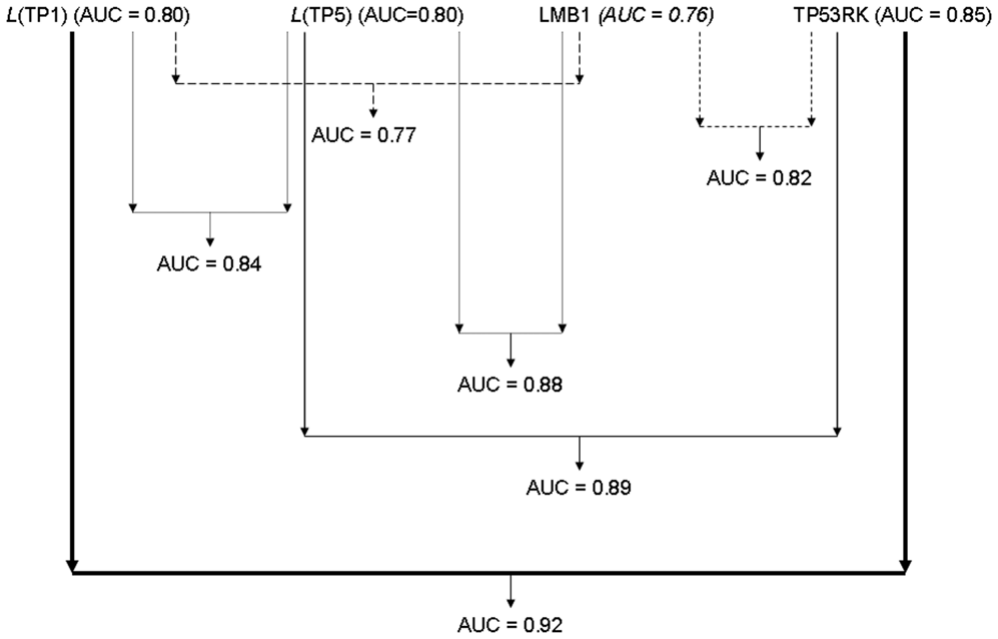
Integrated prognostic systems. Combination of pathway- and gene-based biomarkers for the classification of VD+ and VD− samples. The diagram is read from the top to the bottom, and depicts the predictive capacity estimated when integrating representative differentially expressed genes (LMB1 and TP53RK) and the activity levels of top-perturbed pathways (TP1 and TP5). Only the top single and integrated biomarker models are shown here. Lines linking two biomarkers (genes or pathways) indicate their combination as inputs to an integrated classifier. Different dotted and solid lines are used to facilitate the visualization of the multiple combinations. Arrows indicate the classification performance outcome. AUC: area under the ROC curve. The AUC values obtained with classification models based on single biomarkers are also shown, next to each biomarker and between parentheses.

Figure 6 illustrates how such integration can generate diverse, improved classification outcomes. Only the top single and integrated biomarker models are shown here. The diagram depicts the predictive capacity estimated when integrating representative, differentially expressed genes (LMB1 and TP53RK) and the activity levels of top-perturbed pathways (TP1 and TP5). In Figure 6, lines linking two biomarkers (genes or pathways) indicate that these biomarkers were used as inputs to an integrated classifier. Different dotted and solid lines are used to facilitate the visualization of the multiple combinations. Arrows indicate the classification performance outcome, as estimated with AUC values. When the diagram is read from the top to the bottom, the incremental improvement in classification performance can be visualized for the different input feature combinations. The top classification performance was obtained when the expression values of TP53RK and the *L* values extracted from TP1 (Table 1) were used as inputs to the classification model (AUC = 0.92). These findings provide additional evidence of the potential of our modelling approach for: a) gaining deeper mechanistic insights into processes implicated in (post-MI) ventricular dysfunction, and b) exploring potentially novel biomarker discovery strategies.

### Biological insights

We finally sought to evaluate whether our pathway-driven analysis can provide novel insights into specific molecular and cellular mechanisms involved in ventricular dysfunction after MI. We started by concentrating on LMNB1, one of the top 10 most differentially expressed genes between VD+ and VD− samples, as detected by GSEA. LMNB1 alone provided significant prognostic information (Figure 6). We first confirmed the microarray data with quantitative PCR. Indeed, a significant correlation between both techniques was demonstrated (r = 0.43, P = 0.01, Pearson correlation) and we observed more LMNB1 mRNA in blood cells from VD+ than in those from VD− patients (n = 16 in each group): 0.31±0.14 vs. 0.21±0.09 (mean ± standard deviation, P = 0.01, Mann-Whitney Rank Sum Test). We also compared LMNB1 expression obtained by quantitative PCR between patients with MI and patients with atypical chest pain and without MI. LMNB1 was more expressed in MI patients (n = 22) than in non-MI patients (n = 12): 2.47±1.20 vs. 1.50±0.77 (P = 0.007, Mann-Whitney Rank Sum Test). LMNB1 is the main constituent of nuclear lamina, which participates in the control of gene expression [22]. Interestingly, LMNB1 has been shown to control oxidative stress responses [23]. Therefore, the increase of LMNB1 expression after MI is consistent with activation of oxidative stress. The prognostic performance of LMNB1 has recently been reported for liver cancer [24]. Our study reveals for the first time that LMNB1 may be a potential biomarker in the cardiovascular arena.

One of the pathways highlighted by our pathway-specific activity level analysis was the SIG_REGULATION_OF_THE_ACTIN_CYTOSKELETON_BY_RHO_GTPASES pathway (TP9, Table 1). This pathway involves the regulation of the activation state of cofilin through phosphorylation mediated either by PI3K-RHO-CDC42-PAK-LIMK or PLC-LIMK [25]. There are experimental reasons to believe that this pathway is involved in the development of ventricular dysfunction, and investigations are ongoing in our laboratory to test this hypothesis.

In conclusion, our pathway-driven analyses enabled us to find novel biomarkers and therapeutic targets in the setting of MI and ventricular dysfunction. Studies in larger patient populations are required to examine the clinical usefulness of our findings.

### Shareable software system

The pathway-based modelling approach presented here has been implemented into a computing-platform independent software tool, which is freely available on request from the authors. This demonstration prototype, *kipuMarkers* (for knowledge-driven integrative identification of pathway-based biomarkers), allows users to input a gene expression dataset (samples and class labels) and a list of gene/protein sets that can represent known molecular pathways or other types of biologically-meaningful groups, such as co-regulated genes or sets of miRNA targets. The tool computes *psc* and *P* values for all the user-defined pathways. Moreover, *kipuMarkers* automatically generates a dataset with the samples encoded with their corresponding pathway activity levels. The resulting dataset can afterwards be used to develop different statistical analyses and classification models.

## Discussion

In this investigation we gained new insights into the diversity and extent of potential perturbations observed in post-MI ventricular dysfunction samples across hundreds of molecular pathways. An exploration of such landscape of molecular perturbations reveals the existence of pathways highly-altered between VD− and VD+, which provides not only a system-level mechanistic understanding, but also a foundation for the automated prediction of clinical outcomes. Moreover, this type of research may assist in the identification of feasible therapeutic strategies beyond the single-gene targeting paradigm.

Although the identification of putative biomarkers based on their individual expression patterns will continue to represent a fundamental research tool, there is a need to explore methodologies not only for improving prognostic performance, but also for augmenting our understanding of processes defining the emergence and progression of HF after MI. We showed that powerful and descriptive models of post-MI ventricular dysfunction can be obtained through the incorporation of gene expression and pathway context information during the search for potential biomarkers. This differs with the traditional discovery approach in which gene expression analysis and process descriptions, e.g. GO analysis of differentially expressed genes only, are implemented as separate tasks.

Previous research also suggests that pathway-based signatures may be more reproducible and biologically-interpretable than traditional approaches based on the analysis of the expression of lists of genes [9], [10], [12]. Furthermore, an emphasis on the detection of highly-differentially expressed genes may represent a severe restriction on the identification of genes with potential causal roles in disease [12]. This is because many upstream causal factors may be driven by mutations and events with subtle responses at the transcriptional and post-translational levels.

An important strength of this methodology is that we can identify potential relevant biomarkers and targets, as well as their pathway-based activity signatures, through an unbiased and automated analytical process. We showed how different approaches to estimating pathway-specific activity levels can be used to distinguish between clinical outcomes in post-MI patients. This was illustrated with the unsupervised and supervised analysis of pathway perturbation patterns. Also we explored the potential predictive value of combining pathway-based activity scores and gene expression information obtained from an alternative, published analysis technique. We showed how such computational integration can improve prognostic performance. So far, brain natriuretic peptide (BNP) is considered the golden standard for biomarkers of ventricular dysfunction. However, in our hands, the combination of the pathway TP1 with the gene TP53RK provided a maximal prognostic performance with an AUC of 0.92, which is far better than BNP alone or BNP combined with other markers [26]. Our approach was motivated by the observation that alternative approaches to pathway analysis can provide complementary predictive power and vistas of relevant pathways.

This alternative, yet complementary, prediction capability is a consequence of the methodological differences exhibited by the approaches investigated here. Our approach and GSEA [9] differ with regard to the following aspects: pathway scoring, differential pathway detection, and sample-specific information representation for classification. For example, we estimate pathway activity by calculating the mean expression value observed in a sample, while GSEA focuses on the differential expression values observed at the gene level. Although GSEA exploits pathway-based information to detect relevant genes, this method emphasizes a gene-centric approach to sample representation and classification. Nevertheless, in connection to the identification of highly de-regulated pathways, we also found predictive agreements between these methods. This may be interpreted not only as an indication of the potential relevance of these pathways, but also as supporting evidence for the predictions made by our approach.

Among our significant findings, we found that a pathway responsible for antigen dependent B- cell activation (TP1) could encode a powerful feature to discriminate between VD− and VD+ samples. Note that this pathway does not share genes with the other highly-altered pathways detected by our approach or with the GSEA-derived genes (Figure 4). Such a reduction in predictive information redundancy, together with the strong class discriminatory power of this pathway alone, serve to explain the good prognostic potential of this pathway in conjunction with other potential biomarkers.

The down-regulation of TP1 in patients with VD+ appears plausible since several of its components are known to be protective. For instance, IL10 has been shown to be cerebroprotective in the setting of experimental stroke [27] and soluble receptors of TNF-α limit its cardiotoxicity. The CCR3 receptor has been implicated in allergy but also very recently in the growth of choroidal vessels [28] but its implication in ventricular dysfunction remains to be studied. Perturbation of the HYPERTROPHY_MODEL is also plausible since the development of HF after MI is mediated, at least partly, by a hypertrophy of the left ventricle. Another potentially important pathway highlighted by our analyses is the PELP1PATHWAY. PELP1 is known to serve as an estrogen receptor alpha coactivator with prognostic value for several types of cancer [29], [30] and is linked to the GO biological process “response to hypoxia”.

Perturbation of the VALINE_LEUCINE_AND_ISOLEUCINE_BIOSYNTHESIS and ALANINE_AND_ASPARTATE_METABOLISM pathways suggests a de-regulation of amino acid and protein synthesis as a potential mechanism for ventricular dysfunction Together with the observation that other pathways of energy metabolism are perturbed in VD+ (MPRPATHWAY and ERKPATHWAY), our data suggest that a deregulation of metabolism occurs in some patients after MI.

### Possible limitations

A more conclusive assessment of the potential biomedical relevance of our findings is evidently constrained by the need to perform independent experimental validations. In the future, this can involve the evaluation of the resulting classification models on independent datasets, and the *in vivo* or *in vitro* perturbation of the potentially-significant pathways in case-control samples. The former is directly concerned with the validation of the predictive capacity of the pathway-based patterns as prognostic biomarkers, and the latter will be required to assess the possible therapeutic value of some of these pathways.

Another possible limitation of our investigation is the relatively modest size of the gene expression dataset. Nevertheless, the integrative (pathway and gene expression) analysis strategy proposed here was indeed motivated to address this typical, and probably unavoidable, restriction in proof-of-concept translational research applications. By not relying on a single sparse dataset characterised by thousands of noisy and correlated features, one contributes to the reduction of false positive and negative predictions. There is also a need to incorporate additional evidence regarding pathways and other relevant gene/protein sets. The analysis approaches investigated here enable the straightforward incorporation of additional information, which may be useful to indentify biomarkers or potential therapeutic targets: sets of co-regulated genes, sub-networks of protein-protein interactions, and clusters of genes regulated by a common miRNA or transcription factor. A more comprehensive coverage of (curated) network-based information, together with the availability of new computational tools, will significantly contribute to a more accurate and personalized patient management.

## Supporting Information

Table S1 Detailed functional description of the top 10 molecular pathways exhibiting the largest gene expression perturbations in VD+ samples. BP and CC: Examples of biological processes and cellular localizations highly statistically detectable in a pathway as defined in the GO. P: statistical significance of the GO term over-representation. Up/down indicates the direction of the change in gene expression in the pathway, i.e., “Up” means that the pathway is up-regulated in VD- in comparison to VD+. NS: Non-significant enrichment of terms. TP: Top pathway number.
(0.04 MB DOC)

Table S2 Top 10 perturbed pathways detected by our approach and by GSEA.
(0.02 MB DOC)
