# Supplementary material for: Integrative Pathway-Centric Modeling of Ventricular Dysfunction after Myocardial Infarction
Source: PLoS One. 2010 Mar 11;5(3):e9661. doi: 10.1371/journal.pone.0009661 (PMC2836383; doi:10.1371/journal.pone.0009661)
Supplement: Table S1 — Detailed functional description of the top 10 molecular pathways exhibiting the largest gene expression perturbations in VD+ samples. BP and CC: Examples of biological processes and cellular localizations highly statistically detectable in a pathway as defined in the GO. P: statistical significance of the GO term over-representation. Up/down indicates the direction of the change in gene expression in the pathway, i.e., “Up” means that the pathway is up-regulated in VD- in comparison to VD+. NS: Non-significant enrichment of terms. TP: Top pathway number. (0.04 MB DOC) [file pone.0009661.s001.doc]

Table S1. Detailed functional description of the top 10 molecular pathways exhibiting the largest gene expression perturbations in VD+ samples. BP and CC: Examples of biological processes and cellular localizations highly statistically detectable in a pathway as defined in the GO. *P*: statistical significance of the GO term over-representation. Up/down indicates the direction of the change in gene expression in the pathway, i.e. “Up” means that the pathway is up-regulated in VD- in comparison to VD+. NS: Non-significant enrichment of terms. TP: Top pathway number.

| **TP** | **Name** | **Up/down** | **BP** | **CC** |
| --- | --- | --- | --- | --- |
| TP1 | ASBCELLPATHWAY | Down | lymphocyte activation (*P* = 3.24E-8) | plasma membrane (*P* = 3.76E-3) |
| TP2 | CCR3PATHWAY | Up | intracellular signaling cascade (*P* =1.66E-5) | extrinsic to membrane (*P* = 11E-4) |
| TP3 | PELP1PATHWAY | Up | response to hypoxia  (*P* = 0.019) | NS |
| TP4 | HYPERTROPHY_MODEL | Up | muscle development (*P* = 4.87E-2) | extracellular space (*P* = 3.64E-3) |
| TP5 | VALINE_LEUCINE_AND_ISOLEUCINE_BIOSYNTHESIS | Down | alcohol catabolic process (*P* = 1.55E-3) | mitochondrion (*P* = 7.43E-3) |
| TP6 | METPATHWAY | Up | protein amino acid phosphorylation (*P* =9.07E-7) | leading edge (*P* =14E-2) |
| TP7 | ALANINE_AND_ASPARTATE_ METABOLISM | Down | organic acid metabolic process (*P* =1.13E-18) | Mitochondrion (*P* = 2E-4) |
| TP8 | MPRPATHWAY | Up | phosphate metabolic process (*P* = 6.29E-6) | cAMP-dependent protein kinase complex (*P* = 2.98E-9) |
| TP9 | SIG_REGULATION_OF_THE_ACTIN_ CYTOSKELETON_BY_RHO_GTPASES | Up | cytoskeleton organization and biogenesis (*P* = 1.32E-9) | intracellular non-membrane-bound organelle (*P* = 4.16e-12) |
| TP10 | ERKPATHWAY | Up | phosphate metabolic process (*P* =2.15E-7) | plasma membrane (*P* =1.22E-2) |
