# Supplementary material for: Integrative Pathway-Centric Modeling of Ventricular Dysfunction after Myocardial Infarction
Source: PLoS One. 2010 Mar 11;5(3):e9661. doi: 10.1371/journal.pone.0009661 (PMC2836383; doi:10.1371/journal.pone.0009661)
Supplement: Table S2 — Top 10 perturbed pathways detected by our approach and by GSEA. (0.02 MB DOC) [file pone.0009661.s002.doc]

Table S1. Top 10 perturbed pathways detected by our approach and by GSEA.
